# Retinal Circular RNA hsa_circ_0087207 Expression Promotes Apoptotic Cell Death in Induced Pluripotent Stem Cell-Derived Leber’s Hereditary Optic Neuropathy-like Models

**DOI:** 10.3390/biomedicines10040788

**Published:** 2022-03-28

**Authors:** Yi-Ping Yang, Yuh-Lih Chang, Yun-Hsien Lai, Ping-Hsing Tsai, Yu-Jer Hsiao, Long Hoang Nguyen, Xue-Zhen Lim, Chang-Chi Weng, Yu-Ling Ko, Chang-Hao Yang, De-Kuang Hwang, Shih-Jen Chen, Shih-Hwa Chiou, Guang-Yuh Chiou, An-Guor Wang, Yueh Chien

**Affiliations:** 1 Department of Medical Research, Taipei Veteran General Hospital, Taipei 11217, Taiwan; molly0103@gmail.com (Y.-P.Y.); tony1233000@yahoo.com.tw (Y.-H.L.); figatsai@gmail.com (P.-H.T.); yj1007hsiao@yahoo.com (Y.-J.H.); nguyenhoanglong8888@gmail.com (L.H.N.); susanlimxz@gmail.com (X.-Z.L.); power07272001@hotmail.com (Y.-L.K.); shchiou@vghtpe.gov.tw (S.-H.C.); 2 Institute of Food Safety and Health Risk Assessment, School of Pharmaceutical Sciences, National Yang Ming Chiao Tung University, Taipei 11217, Taiwan; 3 School of Medicine, National Yang-Ming Chiao Tung University, Taipei 11217, Taiwan; m95gbk@gmail.com (D.-K.H.); sjchen96@gmail.com (S.-J.C.); agwang@vghtpe.gov.tw (A.-G.W.); 4 Department of Pharmacy, Taipei Veterans General Hospital, Taipei 11217, Taiwan; ylchang@vghtpe.gov.tw; 5 Department of Pharmacy, National Yang Ming Chiao Tung University, Taipei 11217, Taiwan; 6 Institute of Pharmacology, College of Medicine, National Yang Ming Chiao Tung University, Taipei 11217, Taiwan; 7 Department of Basic Medical Sciences, Hanoi University of Pharmacy, Hanoi 100000, Vietnam; 8 Department of Ophthalmology, Taipei Veterans General Hospital, Taipei 11217, Taiwan; b101097118@gmail.com; 9 Department of Ophthalmology, National Taiwan University Hospital, Taipei 10002, Taiwan; chyangoph@ntu.edu.tw; 10 Department of Ophthalmology, College of Medicine, National Taiwan University, Taipei 11217, Taiwan; 11 Genomic Research Center, Academia Sinica, Taipei 11217, Taiwan; 12 Department of Biological Science and Technology, College of Biological Science and Technology, National Yang Ming Chiao Tung University, Hsinchu 300093, Taiwan

**Keywords:** Leber’s hereditary optic neuropathy, unaffected carrier, hsa_circ_0087207, retinal ganglion cells, induced pluripotent stem cells

## Abstract

**Backgrounds**: Leber’s hereditary optic neuropathy (LHON) is known as an inherited retinal disorder characterized by the bilateral central vision loss and degeneration of retinal ganglion cells (RGCs). Unaffected LHON carriers are generally asymptomatic, suggesting that certain factors may contribute to the disease manifestations between carriers and patients who carry the same mutated genotypes. Methods: We first aimed to establish the iPSC-differentiated RGCs from the normal healthy subject, the carrier, and the LHON patient and then compared the differential expression profile of circular RNAs (CircRNAs) among RGCs from these donors in vitro. We further overexpressed or knocked down the most upregulated circRNA to examine whether this circRNA contributes to the distinct phenotypic manifestations between the carrier- and patient-derived RGCs. **Results**: iPSCs were generated from the peripheral blood cells from the healthy subject, the carrier, and the LHON patient and successfully differentiated into RGCs. These RGCs carried equivalent intracellular reactive oxygen species, but only LHON-patient iPSC-derived RGCs exhibited remarkable apoptosis. Next-generation sequencing and quantitative real-time PCR revealed the circRNA hsa_circ_0087207 as the most upregulated circRNA in LHON-patient iPSC-derived RGCs. Overexpression of hsa_circ_0087207 increased the apoptosis in carrier iPSC-derived RGCs, while knockdown of hsa_circ_0087207 attenuated the apoptosis in LHON-patient iPSC-derived RGCs. Predicted by bioinformatics approaches, hsa_circ_0087207 acts as the sponge of miR-665 to induce the expression of a variety of apoptosis-related genes in LHON patient iPSC-derived RGCs. **Conclusions**: Our data indicated that hsa_circ_0087207 upregulation distinguishes the disease phenotype manifestations between iPSC-derived RGCs generated from the LHON patient and carrier. Targeting the hsa_circ_0087207/miR-665 axis might hold therapeutic promises for the treatment of LHON.

## 1. Introduction

Leber’s hereditary optic neuropathy (LHON) is an inherited retinal disease named by Theodore Leber, a German ophthalmologist who diagnosed and described this pathology in his patients in 1871 [1]. This inherited retinal disease can cause the damage of retinal ganglion cells (RGCs) that eventually leads to blindness due to the dysfunction of maternally-inherited mutated mitochondria [2]. This vision loss of LHON patients develops particularly in male teenagers with an onset age between 10–30 years old. As soon as one side of the eye turns blind, the other side of the eye will suffer a similar pathology within two months. Epidemiological studies show a prevalence of 1 in 31,000, 1 in 39,000, and 1 in 50,000 in the northern UK, the Netherlands, and Finland, respectively [3]. In addition, LHON is the most common neuropathy caused by primary mitochondria mutation, specifically at G11778A/ND4, G3460A/ND1, and T14484C/ND6 of the mitochondrial genes [4,5]. Among these mutations, ND4 mutation remains the most novel cause of LHON as reported worldwide [3]. Since the discovery of LHON, researchers have studied its mechanisms through translational medicine, stem cells differentiation, and gene therapy. However, there is still a distance for researchers to discover the underlying mechanisms and develop an effective treatment for LHON [6].

The mitochondria DNA (mtDNA) of LHON patients have been frequently diagnosed with a homoplasmic primary mutation, which indicates nearly 100% mutant [7]. The single point mutation in the mtDNA takes place in the mitochondrial complex I, the NADH ubiquinone oxidoreductase complex. The most common mutation occurs at position 11778 and causes a change in the amino acid from guanine to adenosine, resulting in mitochondrial complex I dysfunction. As the mitochondrial complex I serves crucial roles in mitochondrial function, its mutation leads to the energy depletion in the neurons that causes the death of RGCs, hence causing blindness in affected patients [8]. Optic neuropathy of mitochondrial dysfunction also causes other diseases such as autosomal dominant optic atrophy (DOA) and Wolfram syndrome [9]. In the RGCs of LHON patients, the mitochondria complex I is affected due to the G11778A point mutation. As a result, the NAD+/NADH ratio decreases and severely impacts mitochondrial function [10]. Abnormal opening of mitochondria pore has been studied and proposed as one of the disease mechanisms, and mitochondria complex I deficiency has also been shown to increase mitochondria permeability transition pore (mPTP) [11].

In recent years, due to the high-throughput sequencing analysis and bioinformatic methods, circular RNAs (circRNAs) have been discovered and gained wide attention and drove researchers to investigate their roles [12]. CircRNAs are shown to be involved in regulating gene expression through suppressing miRNA [13]. Studies have reported that circRNAs also play other roles in fundamental cellular functions, such as transcription regulation, m^6^A translation, cell cycle regulation, and protein sponge [14]. Unlike canonical splicing on a linear messenger RNA (mRNA), circRNA biogenesis involves a back-splicing that, after introns are being spliced away, one end of the exon joins with the other end of exon or even intron [15], resulting in the formation of the stable circular structure with a back-spliced junction. In brief, the functional roles of circRNAs shown in various human diseases such as neurodegenerative diseases and cancer [16,17] elicit a great potential for circRNAs in clinical applications. The unique structure, high stability, and abundant presence of circRNAs make them potential biomarkers for diagnosing and predicting diseases. Hence, this study aims to investigate the differential expression profile of circRNAs involved in iPSCs-differentiated RGCs and further investigate the role of circRNAs that may be involved in the pathogenesis of LHON disease.

Compared with LHON patients, unaffected carriers are generally asymptomatic, suggesting that certain additional factors rather than the ND4 mutation may distinguish the disease manifestations in patients and carriers with the same mutated genotype. In this study, we first established the iPSC-differentiated RGCs from the normal healthy subject, the carrier, and the LHON patient, and then compared the differential expression profile of circRNAs among RGCs from distinct origins in vitro. We identified the most upregulated circRNA in the LHON patient-derived RGCs compared to that in the carrier-derived group.

## 2. Materials and Methods

### 2.1. Derivation of Human Induced Pluripotent Stem Cells (iPSCs)

Human iPSCs were derived from peripheral blood mononuclear cells (PBMCs) of the family members of the LHON patient, including the normal healthy subject (father), the LHON carrier (mother), and the LHON patient (son). The inclusion criteria for the affected LHON subject are a confirmed genotypic diagnosis of LHON, supported by visual function outcome data including at least two visual function assessments between one year and three years after vision loss. The exclusion criteria of affected LHON subject is those who have received any investigational drug. A healthy subject, an unaffected carrier, and the LHON patient from the same family were enrolled in this study. Ethical approvals from the Institutional Review Board on Biomedical Science Research at Taipei Veterans General Hospital Institutional Review Board (2016-03-003A) were attained. PBMCs of 5 × 10^5^ were seeded on a 24-well plate and were maintained in StemPro^®^-34 medium (Gibco, Grand Island, NY, USA) supplemented with 100 ng/mL SCF, 100 ng/mL FLT3, 20 ng/mL IL-3, and 20 ng/L IL-6 for four days. Cells were then reprogrammed by the infection with Sendai virus expressing four Yamanaka factors, OCT4, SOX2, KLF4, and c-MYC, using the CytoTune^®^-iPS 2.0 Sendai Reprogramming Kit (A16518, Thermo Fisher Scientific, Waltham, MA, USA) following the manufacturer’s instruction. Twenty-four hours later, PBMCs were harvested and cultured in 1 mL of fresh PBMC complete medium for two days, then transferred to MEF feeders and maintained in StemPro^®^-34 medium without cytokines. The medium was changed every two days. Seven days after infection, the culture medium was replaced with human embryonic stem cells (hESCs) medium containing DMEM/F12 with 20% Knock-out Serum Replacement (KOSR), 1 mM L-glutamine, 0.1 mM Non-essential amino acids, 55 µM 2-mercaptoethanol, and 10 ng/mL bFGF, and were incubated in a humidified atmosphere of 95% air and 5% CO_2_ at 37 °C. The medium was changed every day. After the procedures, multiple iPSC colonies were generated and assigned for further examination.

### 2.2. Differentiation of iPSCs into Retinal Ganglion Cells (RGCs)

After reaching confluency, iPSCs cultured in StemFlex medium were detached enzymatically by versene and dissociated into single cells. These cells were then transferred to a non-adherent culture dish with StemFlex and neural induction media (NIM) medium containing Advanced DMEM/F12, non-essential amino acid, 1% N2 supplement (Invitrogen), and 2 mg/mL heparin (Sigma, Saint Louis, MO, USA) to induce the formation of embryoid bodies (EB). The iPSCs were then incubated with 10 µM of Rho-associated protein kinase (ROCK) inhibitor Y-27632 to increase the cell viability. The medium was renewed every other day with the ratio of 3:1 StemFlex/NIM, 1:1 StemFlex/NIM, 1:3 StemFlex/NIM, and 100% NIM. Neural rosettes were formed after 1 week of culture. On day 17, cells were cultured with retinal differentiation media (RDM) containing Advanced DMEM/F12 (3:1), supplemented with 2% B27 (with Vitamin A, Invitrogen, St. Louis, MO, USA) and non-essential amino acid for 10 consecutive days. Neurospheres were observed and transferred to laminin-coated dishes for RGC differentiation. Forty-five days after the differentiation, the growth of neurite can be reproducibly observed.

### 2.3. Patch-Clamp Analysis

RGCs were attached to the coverslips and stabilized in the incubating chamber. After the incubation, the coverslips were immersed with ACSF: 125 mM NaCl, 2.5 mM KCl, 25 mM NaHCO_3_, 1.25 mM NaH_2_PO_4_, 2 mM CaCl2, 1 mM MgCl_2_, and 25 mM glucose (pH 7.4 with carbogen) in the recording chamber. Neurons were visualized by infrared differential interference contrast (DIC) microscopy. The recording pipettes (8–10 MΩ) were filled with internal solution containing the following constituents: 135 mM K-gluconate, 20 mM KCl, 2 mM MgCl_2_, 10 mM HEPES, 0.1 mM EGTA, and 4 mM Na_2_ATP (pH 7.3). Signals were recorded by MultiClamp 700B amplifiers (Molecular Devices, Sunnyvale, CA, USA). Data were filtered at 2 kHz and sampled at 10 kHz with a Digidata 1440A interface (Molecular Devices) controlled by pCLAMP version 10.6 (Molecular Devices).

### 2.4. Reactive Oxygen Species (ROS) Assay

ROS production was determined using 2′, 7, -dichlorodihydrofluorescin diacetate (DCFH-DA) staining assay (Abcam, Cambridge, UK). Cells were incubated and stained with DCFH-DA for 30 min and were resuspended in PBS before the analysis using flow cytometry.

### 2.5. Measurement of Apoptosis

Annexin V/PI double-staining detection kit was used to detect the percentage of cells under viable, necrotic, or apoptotic status. RGC cells from the normal healthy subject, the carrier, and LHON patient were stained with annexin-V-propidium iodide (PI) labeling solution in the dark. The collected cells were analyzed using a flow cytometer.

### 2.6. Immunofluorescence Assay

Cells were seeded on 24-well plates until confluence. After reaching the confluence, the medium was discarded, and cells were washed with PBS. Cells were then fixed in 4% paraformaldehyde for 10 min at RTP. Paraformaldehyde was discarded and the cells were permeabilized with 0.1% Triton X-100 in PBS for 10 min. Cells were then washed twice with PBS and blocked in a blocking solution of 5% FBS in PBS for 30 min. Primary antibody was added to the cells at respective dilution in PBS buffer containing 1% FBS and was kept overnight at 4 °C. Cells were washed twice with PBS before incubating in the dark with FITC-labelled secondary antibody in PBS buffer containing 1% FBS for 1 h. The solution was aspirated and washed trice with PBS. Nuclear DNA was stained with Hoechst at a dilution of 1:1000 in PBS buffer containing 1% FBS for 10 min. Cells were washed trice with PBS solution before the microscopical examination.

### 2.7. RNA Extraction and Quantitative Real-Time Polymerase Chain Reaction (qRT-PCR)

RNA extraction was conducted using TRIzol reagent (Invitrogen Life Technologies) according to the manufacturer’s protocol. The concentration and sample quality of the extracted RNA was determined by NanoDrop. Complementary DNA (cDNA) was synthesized from 5 µg of extracted RNA using SuperScript III Reverse Transcriptase (Invitrogen) and other buffers, after which cDNA was diluted 20-fold before the subsequent PCR amplification with SYBR^®^qPCR Master Mix kits. The qRT-PCR was performed on the StepOnePlusTM Real-Time PCR System (Applied Biosystems, Foster City, CA, USA) with MicroAmp FAST 96-Well Reaction Plate (Applied Biosystems, Foster City, CA, USA). The primers used for qRT-PCR are listed in Table 1.

### 2.8. Data Processing, Functional Enrichment Analysis, and Network Analysis

As the microarray result, we used Affymetrix U133 plus 2.0 to compare the different expressions among the normal, carrier, and patient samples. Then, we used R language (R 4.1.2) and applied the package “affy” to quantify the expression and normalize with “rma” function. After normalization, we used Excel to calculate the fold change and to sort the most affected genes. Heatmap was conducted with Orange version 2.7 (https://orangedatamining.com/; version 2.7, Ljubljana, Slovenia; accessed date: 25 May 2013). Gene Ontology (GO) pathway enrichment analyses were conducted using Enrichr (http://amp.pharm.mssm.edu/Enrichr/; orginal version; New York, USA; accessed date: 29 March 2021) for clusters obtained from each microRNA target; meanwhile, we only used the upregulated targets for pathway prediction. Enrichr is a web-based tool that allows the evaluation of annotations with its extensive gene-set libraries. The GO Biological Process 2018 of each tissue was determined. The twelve most significant GO biological processes of miR-665 are selected with the threshold of adjusted *p*-value < 0.05 and odds ratio > 0. The search tool for retrieval of interacting genes (STRING) database (https://string-db.org; version 11.0, Heidelberg, Germany; accessed date: 19 January 2019), which integrates both known and predicted protein–protein interaction and transcription regulation, can be applied to predict functional interactions of miRNA targets [10.1093/nar/gku1003]. To seek potential core regulators among the miRNA targets, the STRING tool was employed.

## 3. Results

### 3.1. Generation of the iPSC-Derived RGCs from the LHON Patient and His Unaffected Family Members

**Figure 1:**
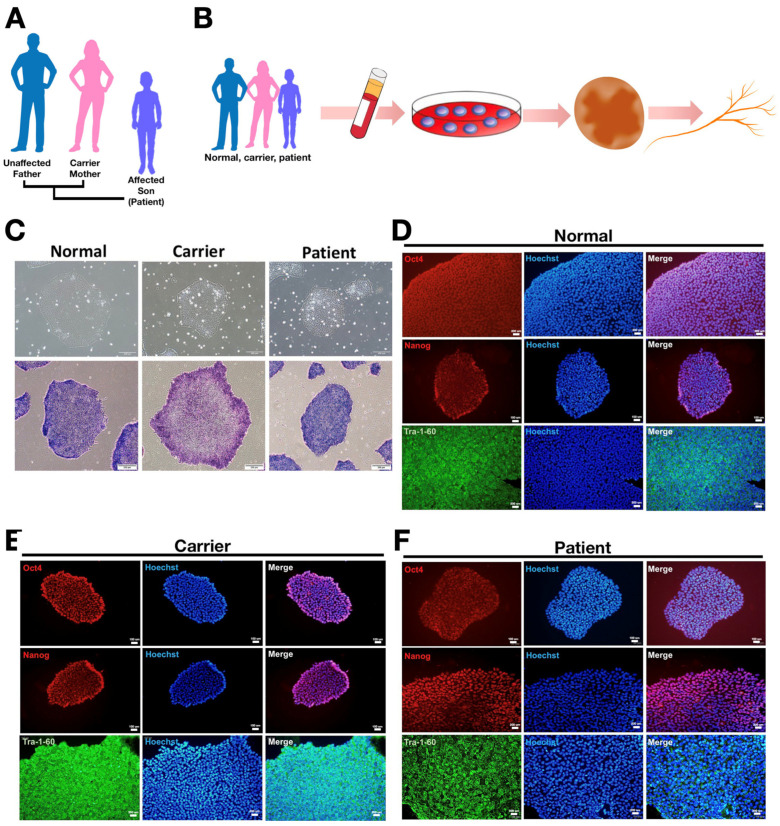
Characterization of PBMC-derived iPSCs from the LHON patient and his family members. (**A**) the family pedigree chart of the enrolled LHON patient; (**B**) schematic illustrations of the generation of RGCs from the enrolled LHON patient and his family members; (**C**) upper: Bright-field images of PBMC-derived iPSCs; lower: Alkaline phosphatase staining revealed the typical pluripotency property of iPSCs. Scale bar: 100 μM (×20). Immunofluorescence staining showed high expression of stemness marker Oct-4, Nanog, and Tra-1-60 in (**D**) the normal subject-derived iPSCs; (**E**) carrier-derived iPSCs; and (**F**) LHON patient-derived iPSCs.

CircRNAs have been shown to be involved in various human diseases such as neurodegenerative diseases and cancer [16,17]. The unique structure and high stability of circRNAs render them potential biomarkers for clinical application. Prior to comparing the circRNA expression profiles in iPSC-derived retinal ganglion cells, we generated iPSCs from PBMCs obtained from three individuals of the same family: the normal (father), the LHON carrier (mother), and the LHON patient (son) (Figure 1A). All PBMCs were reprogrammed into iPSCs by ectopic expression of Sendai reprogramming vectors including Oct4, Sox2, Klf4, and c-Myc, which are the core pluripotency-related transcription factors (Figure 1B). Figure 1C shows the morphology of reprogrammed-iPSCs at day 7 of culture. The mature iPSC colonies appeared round and smooth-edged and had a high nucleus-to-cytoplasmic ratio. To confirm the pluripotency of PBMC-derived iPSCs, we evaluated it by the staining of alkaline phosphatase (Figure 1C, lower). In addition, the stemness of iPSCs was also validated by staining the pluripotency-associated cell surface markers, including Oct4, Nanog, and Tra-1-60 (Figure 1D–F). Collectively, the embryonic stem cell-like morphologies, and the pluripotent and stemness characteristics have validated the successful generation of iPSC clones from normal healthy subjects, unaffected carriers, and LHON patients.

**Figure 2:**
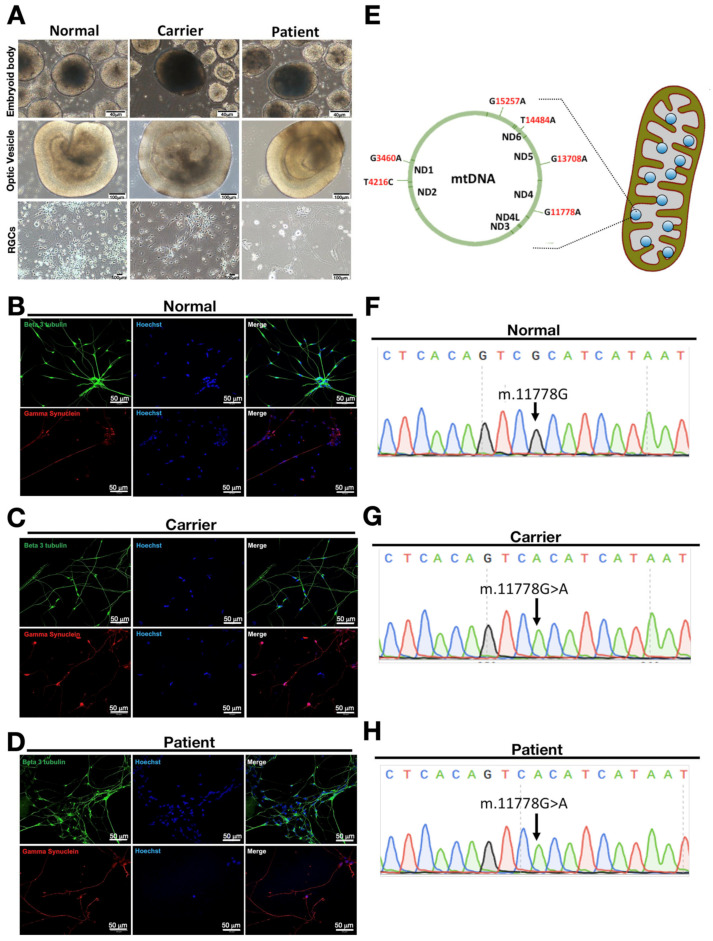
Characterization of iPSC-derived RGCs generated from the LHON patient and his family members. (**A**) Following the stepwise differentiation protocol, healthy donor-derived iPSCs, carrier-derived iPSC, and the LHON patient-derived iPSCs were differentiated. For all clones from distinct origins, the morphology of embryoid bodies (EBs) showed bolus formation at D15, optic vesicles (OV) showed neuronal rosettes at the outer layer at D24, and RGCs at D35 showed neurite outgrowth. Immunofluorescence staining of (**B**) normal iPSC-derived RGCs; (**C**) carrier iPSC-derived RGCs; and (**D**) patient iPSC-derived RGCs showed expression of neuronal markers beta-3 tubulin and gamma synuclein; (**E**) illustration of mitochondrial mutations in LHON disease; (**F**) Sanger sequencing of normal iPSC-derived RGCs without mitochondrial mutation. Sanger sequencing of (**G**) carrier iPSC-derived RGCs and (**H**) patient iPSC-derived RGCs with a mitochondrial mutation at mt.11778G > A (ND4). Panel A, scale bars of embryonic bodies = 40 µm, optic vesicles and RGCs = 100 µm; Panels B, C, and D, scale bars = 50 µm.

LHON is an inherited neuropathy characterized by the significant loss of retinal ganglion cells (RGCs) in the eyes. Therefore, to compare the differential expression profiles of circRNAs in iPSC-derived RGCs from the healthy donor, carrier, or the LHON patient, we next subjected these iPSCs derived from the family members of the LHON patient to the differentiation into RGCs. The embryoid bodies (EBs) were shifted to the neuronal induction medium for seven days and were induced to adhere by the addition of 10% fetal bovine serum, and maintained in the neuronal induction medium for another eight days (Figure 2A). At post-induction day 16, the optic vesicles (OVs) were formed with obvious neuronal rosettes at the outer layer (Figure 2A). On day 25, mature OVs were filtered and dissociated single RGCs were obtained. After seven days, neurite formation was remarkable, and the morphology of RGCs was recorded (Figure 2A). Beta-3 tubulin and gamma synuclein play crucial roles in proper axon guidance and neurofilament network integrity, and both of them are essential for neuronal cytoskeleton formation. We also found these dissociated single RGCs were stained positive for beta-3 tubulin and gamma synuclein, validating proper differentiation from corresponding iPSCs into mature iPSCs (Figure 2B–D).

LHON patients are reported to carry the mitochondria DNA (mtDNA) mutation at multiple points, most commonly G11778A (ND4), G3460A (ND1), and T14484A (ND6) [18]. Apart from these common mutation points, there are other mtDNA alterations that were found to be associated with this disease, such as T4216C, G13708A, and G15257A (Figure 2E). In order to elucidate the mitochondria mutation that occurred in the LHON patient enrolled in this study, mtDNA was extracted from these iPSC-derived RGCs and analyzed by Sanger sequencing. RGCs isolated from the normal healthy donor (the father; Figure 2F) carry nucleotide guanine at mtDNA position 11778, while the guanine nucleotide was mutated at the same position, also known as the putative NADH dehydrogenase 4 (ND4) mutation, in both carrier iPSC- and patient iPSC-derived RGCs (Figure 2G,H). Collectively, our data revealed that iPSCs generated from the carrier and the LHON patient who carry the guanine to adenine mutation are capable of differentiation into mature RGCs.

### 3.2. Assessment of the Phenotypes of iPSC-Derived RGCs from the LHON Patient, the Carrier, and the Healthy Subject

**Figure 3:**
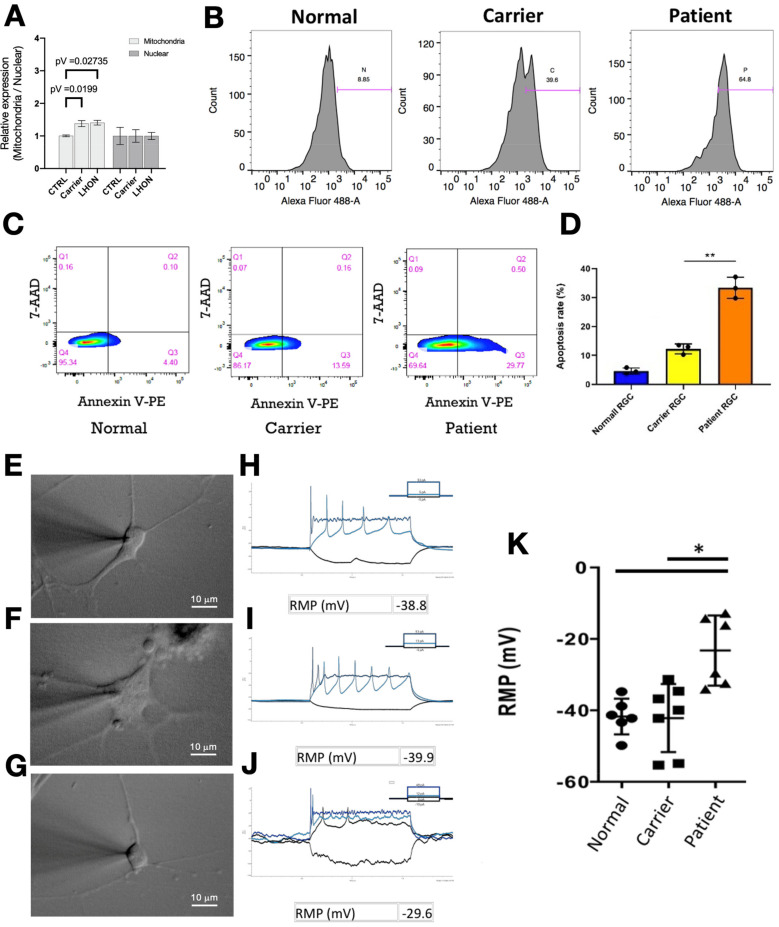
Assessment of the phenotypes of iPSC-derived RGCs from the LHON patient, unaffected carrier, and normal subject. (**A**) Quantitative real-time PCR was performed to analyze the copy number of mitochondrial or nuclear DNA in iPSC-derived RGCs from the LHON patient, unaffected carrier, and normal subject. The copy number of mitochondrial DNA was significantly increased in both carrier- and LHON patient iPSC-derived RGCs, compared to that of the iPSC-derived RGCs from the normal healthy subject. (**B**) Flow cytometry analysis was performed by staining with DCFDA dye to measure the ROS production in iPSC-derived RGCs from the LHON patient, unaffected carrier, and normal subject. A remarkable increase in the ROS levels was detected in the LHON patient iPSC-derived RGCs. (**C**) Apoptosis was measured by using annexin V and propidium iodide (PI) staining in indicated iPSC-derived RGCs; (**D**) the quantification of apoptosis rate from flow cytometry data of indicated iPSC-derived RGCs; (**E**–**G**) morphology and the firing patterns of iPSC-derived RGCs under the patch-clamp recording. Patient iPSC-derived RGCs showed abnormal firing patterns as compared to normal and carrier RGCs. (**H**–**J**) Patch-clamp analysis revealed the resting membrane potential (RMP) levels among the iPSC-derived RGCs from the LHON patient, the carrier, and the normal subject. (**K**) Patch-clamp analysis revealed higher RMP levels in the patient iPSC-derived RGCs. * *p* < 0.05; ** *p* < 0.01. Panels E, F, and G, scale bars = 10 µm.

After the generation of LHON patient iPSC-derived RGCs, we next examined the copy number of mitochondria DNA and the generation of intracellular reactive oxygen species (ROS), electrophysiology performance, and the number of apoptotic cells. As examined by quantitative real-time PCR, the copy number of mitochondria DNA was significantly increased in both carrier- and LHON patient iPSC-derived RGCs, compared to that of the iPSC-derived RGCs from the normal healthy subject (Figure 3A). We then analyzed the reactive oxygen species (ROS) level by DCFH-DA staining. Compared with the iPSC-derived RGCs from the normal healthy subject, the iPSC-derived RGCs from the carrier and the LHON patient both exhibited significant elevation in the intracellular ROS levels (Figure 3A,B). In addition, we carried out apoptosis analysis by staining the RGCs with Annexin-V and propidium iodide (PI). Remarkably, LHON patient iPSC-derived RGCs exhibited a significant increase in apoptotic cell death, compared with that of the normal healthy subject and LHON carrier (Figure 3C,D). To further evaluate and compare the electrophysiological properties among iPSC-derived RGCs generated from the normal healthy subject, the carrier, and the LHON patient (Figure 3E–G), we performed patch-clamp analysis on dissociated single RGCs generated from different donors. Compared with that of the normal healthy subject or LHON carrier, the neuron firing pattern is irregular and incomplete in the iPSC-derived RGCs of the LHON patient (Figure 3H–J). The resting membrane potential (RMP) was significantly higher in the RGCs generated from the LHON patient than that from the healthy subject and carrier (Figure 3K). Taken together, our data demonstrated that both of the iPSC-derived RGCs from the LHON carrier and the patient exhibited high levels of intracellular ROS accumulation. Remarkably, among these RGCs from different donors, only LHON patient iPSC-derived RGCs showed abnormal electrophysiology and high levels of apoptosis.

### 3.3. Identification of Potential circRNA Candidates That Contribute to the Abnormal Phenotypes in LHON Patient iPSC-Derived RGCs

**Figure 4:**
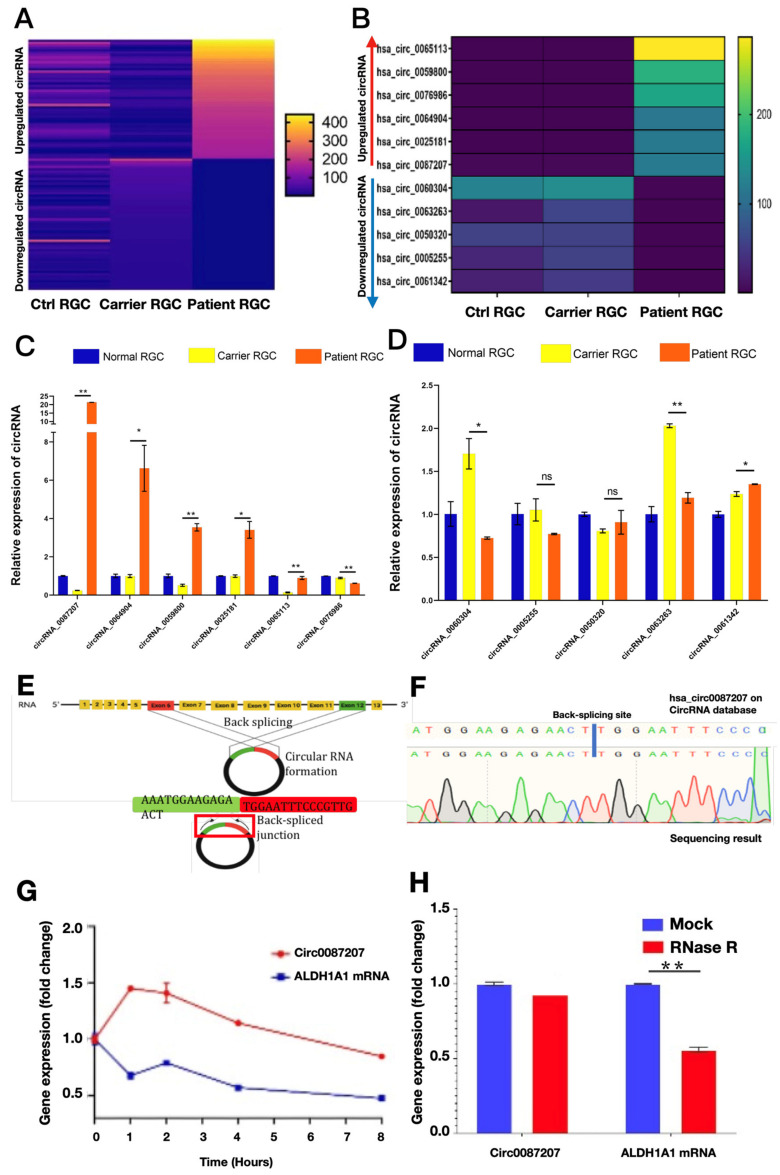
Identification of upregulated circular RNAs in LHON patient iPSC-derived RGCs. (**A**) Heatmap analysis showed the circRNA profiling among the iPSC-derived RGCs generated from the control healthy subject, the carrier, and the LHON patient. (**B**) The top six upregulated circRNA and the top five downregulated circRNAs in LHON patient iPSC-derived RGCs were picked up and shown. The circRNA expression levels of the LHON patient iPSC-derived RGCs were compared with that of the carrier iPSC-derived RGCs. The next-generation sequencing results of (**C**) the most upregulated circRNAs and (**D**) the most downregulated circRNAs in LHON patient iPSC-derived RGCs were validated using quantitative real-time PCR. Among all the upregulated circRNAs, hsa_circ_0087207 exhibited the highest expression in the LHON patient iPSC-derived RGCs as compared with the carrier iPSC-derived RGCs. (**E**) The schematic diagram presented the formation of circ0087207 from the 6th and 12th exons of ALDH1A1 gene. (**F**) The sequence of the identified circRNA with the highest expression matched the sequence and back-splicing junction site of hsa_circ_0087207 published in the online circRNA library on Circular RNA Interactome. (**G**) Actinomycin D assay and (**H**) RNase R assay were used to examine the stability of hsa_circ_0087207. * *p* < 0.05; ** *p* < 0.01. ns *p* > 0.05.

Since circRNAs may participate in the pathogenesis of neurodegenerative diseases and the circular and stable structure of circRNA provide the opportunity for using circRNAs as potential biomarkers for diagnosis or treatment in clinical applications [19]. Accordingly, we carried out next-generation sequencing (NGS) to analyze the circRNA profiling among iPSC-derived RGCs generated from the normal healthy subject, the carrier, and the LHON patient. Compared with that of the healthy subject or LHON carrier, NGS results indicated that several circRNAs were upregulated or downregulated in the iPSC-derived RGCs generated from the enrolled LHON patient, compared with that of the iPSC-derived from the carrier (Figure 4A). The top six of the most upregulated circRNAs and the top five of the most downregulated circRNAs were picked up for further validation and analysis (Figure 4B). We next validated the results of next-generation sequencing using quantitative real-time PCR. Among all the top six upregulated circRNAs, hsa_circ_0087207 is the most upregulated circRNA in the iPSC-derived RGCs generated from the LHON patient, compared with that from the healthy subject or LHON carrier (Figure 4C). Among the five downregulated circRNAs in patient iPSC-derived RGCs, only the expression of hsa_circ_006004 and hsa_circ_0063263 fitted the next-generation sequencing prediction and were lower than that of carrier-derived RGCs (Figure 4D). Instead of the canonical splicing pathway, the biogenesis of circRNA occurs through the back splicing of exons. The joining of both exons forms a back spliced junction with a unique sequence that is not found in linear RNA. The formation of hsa_circ_0087207 occurs from the 6th and 12th exons of ALDH1A1 gene (Figure 4E). Sanger sequencing further validated that the sequence of our identified circRNA with the most upregulated expression matched the sequence and the back-splicing junction site of hsa_circ_0087207 published in the online circRNA library on Circular RNA Interactome (https://circinteractome.nia.nih.gov/; original version, Maryland, USA; accessed date: 30 January 2020) (Figure 4F). Since circRNAs have been known to carry high stability than linear RNAs, we then analyzed the stability of hsa_circ_0087207 in LHON patient iPSC-derived RGCs using the actinomycin D assay. After the treatment of LHON patient iPSC-derived RGCs with actinomycin D, the half-life of the hsa_circ_0087207 transcript was longer than that of linear ALDH1A1 transcript, validating the higher stability of hsa_circ_0087207 than that of ALDH1A1 mRNA in patient iPSC-derived RGCs (Figure 4G). We further conducted the RNase R digestion assay and found that hsa_circ_0087207 was resistant to the treatment of RNase R, a 3′ to 5′ exoribonuclease. Unlike the hsa_circ_0087207, the linear ALDH1A1 mRNA was largely degraded (Figure 4H). These data validated the circular form of hsa_circ_0087207 and its higher stability than the linear mRNAs.

### 3.4. High circ0087207 Levels Contribute to Apoptosis but Not Intracellular ROS Accumulation in LHON Patient iPSC-Derived RGCs

**Figure 5:**
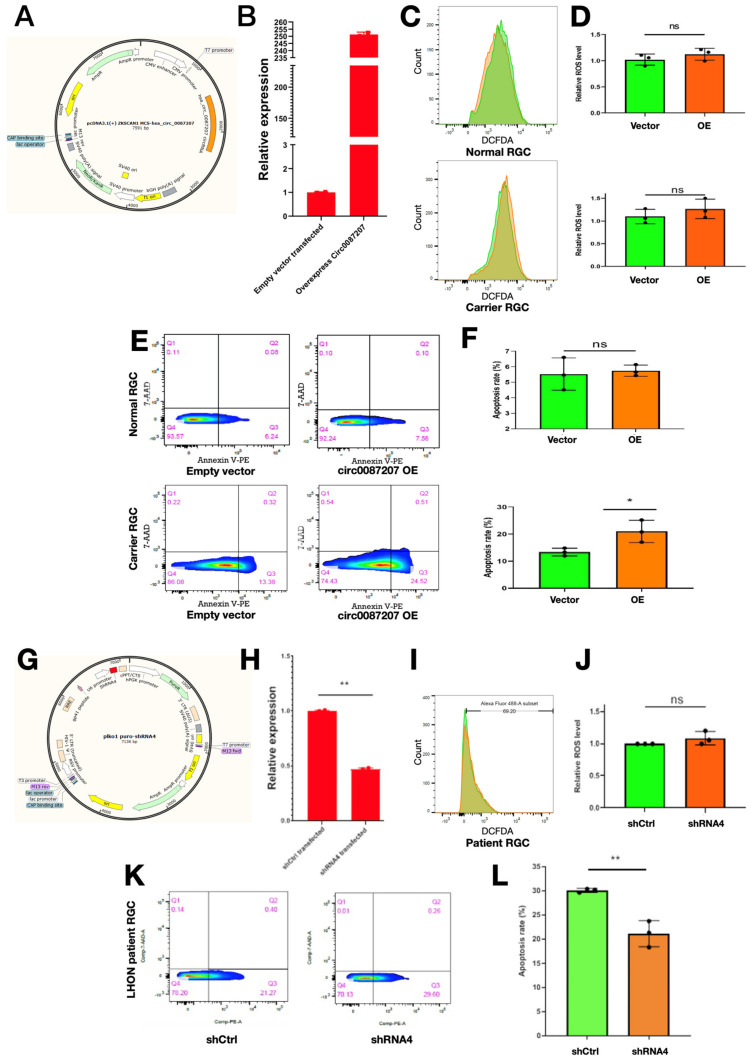
Manipulation of circ0087207 modulated apoptosis but not intracellular ROS levels in LHON patient iPSC-derived RGCs. (**A**) Map of the plasmid pcDNA3.1(+) ZKSCAN1 MCS hsa_circ_0087207 for circRNA_0087207 overexpression; (**B**) validating the overexpression of hsa_circ_0087207 using quantitative real-time PCR. (**C**) Flow cytometry and (**D**) its quantification showed the intracellular ROS accumulation in normal iPSC-derived RGCs (upper) and carrier iPSC-derived RGCs (lower) after hsa_circ_0087207 overexpression. (**E**) Flow cytometry and (**F**) its quantification showed the apoptosis rate in normal iPSC-derived RGCs (upper) and carrier iPSC-derived RGCs (lower) after hsa_circ_0087207 overexpression. (**G**) Map of the plasmid plko1 puro-shRNA4 hsa_circ_0087207 used for circ0087207 knockdown; (**H**) validating the knockdown of hsa_circ_0087207 using quantitative real-time PCR. (**I**) Flow cytometry and (**J**) its quantification showed the intracellular ROS accumulation in LHON patient iPSC-derived RGCs after hsa_circ_0087207 knockdown. (**K**) Flow cytometry and (**L**) its quantification showed the apoptosis rate of LHON patient iPSC-derived RGCs after hsa_circ_0087207 knockdown. * *p* < 0.05; ** *p* < 0.01. ns *p* > 0.05.

Based upon the observations of high circ_0087207 expression in LHON patient iPSC-derived RGCs, we sought to investigate if the high circ_0087207 levels serve a role in LHON disease manifestations. First, we overexpressed hsa_circ0087207 in carrier iPSC-derived RGCs and normal iPSC-derived RGCs and examined the consequences after this overexpression. The plasmid pcDNA3.1(+) ZKSCAN1 MCS hsa_circ_0087207 was used for circ_0087207 overexpression (Figure 5A). Quantitative real-time PCR validated the drastic elevation of circ_0087207 after the transfection of the plasmid (Figure 5B). After the overexpression of circ_0087207, we used flow cytometry to examine the intracellular ROS levels and apoptosis in carrier iPSC-derived RGCs and normal iPSC-derived RGCs. Overexpression of circ0087207 did not modify the intracellular levels of ROS in both normal iPSC-derived RGCs (Figure 5C, upper and Figure 5D, upper) and carrier iPSC-derived RGCs (Figure 5C, lower and Figure 5D, lower). Remarkably, overexpression of circ_0087207 did not affect the basal apoptosis rate in normal iPSC-derived RGCs (Figure 5E, upper; Figure 5F. upper) but increased apoptosis in carrier iPSC-derived RGCs (Figure 5E, lower; Figure 5F, lower). On the contrary, considering the observations of high expression of circ0087207 in patient iPSC-derived RGCs, we also attempted to knock down circ0087207 expression in patient iPSC-derived RGCs and examine the knockdown effect on apoptosis and intracellular ROS levels in such cells. The plasmid plko1 puro-shRNA4 hsa_circ_0087207 was used for circ0087207 knockdown (Figure 5G) and the knockdown effect on circ_0087207 expression was examined by quantitative real-time PCR (Figure 5H). Flow cytometry was used to evaluate intracellular ROS levels and apoptosis. Overall, circ_0087207 knockdown did not affect the intracellular ROS levels (Figure 5I,J) but significantly ameliorated apoptosis in LHON patient iPSC-derived RGCs (Figure 5K,L). Taken together, the findings of overexpression and knockdown studies indicated a crucial role of circ_0087207 that contributes to apoptosis in LHON patient iPSC-derived RGCs.

### 3.5. The circ_0087207/miR-665 Axis Is Predicted to Exert Noxious Effects in LHON Patient iPSC-Derived RGCs

**Figure 6:**
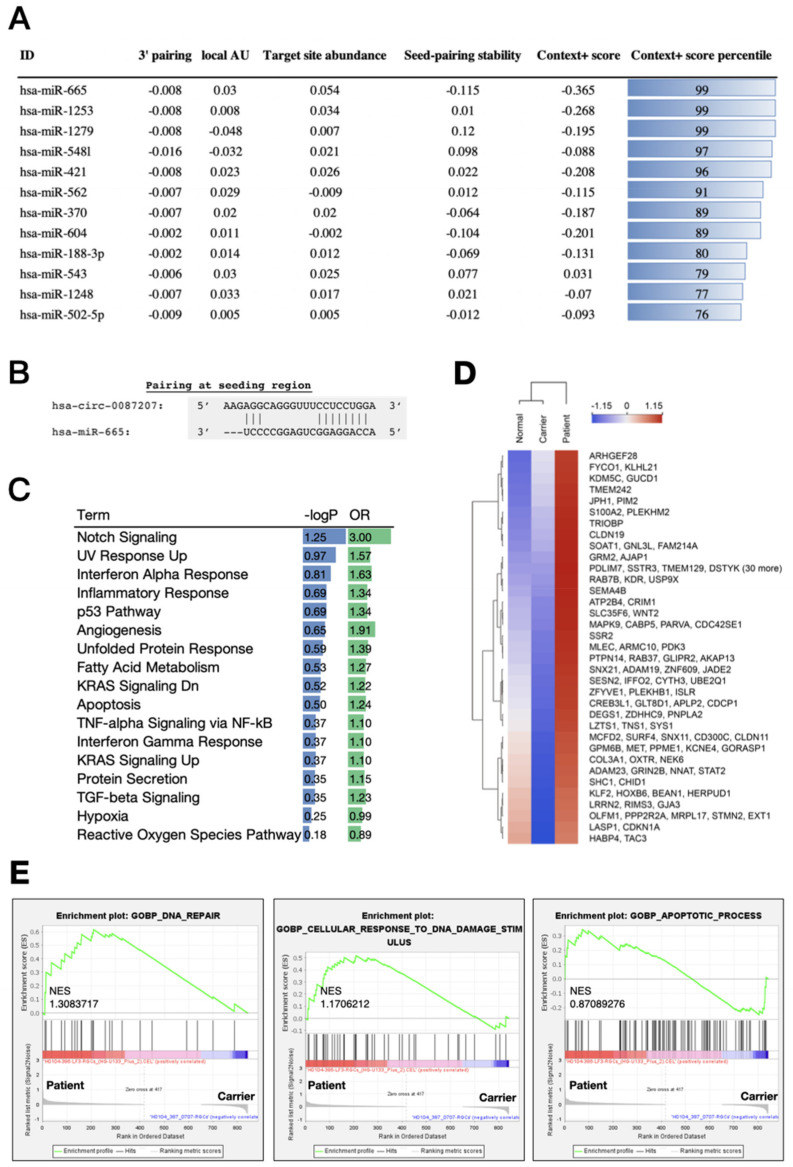
Bioinformatic characterization of miR-665 targets in RGC cells. (**A**) the theoretical affinity of each microRNA to hsa_circRNA_0087207; (**B**) CircRNA-miRNA interactions identified by the public databases CircInteractome. hsa_circRNA_0087207/hsa-miR665; (**C**) gene ontology term of the miR-665 targets. −log*p*: −log(*p*-value). OR: odds ratio; (**D**) hierarchical clustering heatmap of the 350 genes with the largest variance. The expression of genes among normal, carrier, and patient sample were log transformed and z-score normalized across samples. Green indicates low expression and red high; (**E**) gene set enrichment analysis (GSEA). GSEA was performed in the LHON carrier and patient RGC cells. The GSEA algorithm calculates an enrichment score indicating the degree of overrepresentation at the top or bottom of the ranked list of the genes included in a gene set in a ranked list of all genes present in the microarray dataset. A positive normalized enrichment score (NES) indicates gene set enrichment at the top of the ranked list. The analysis demonstrates that (left) GOBP DNA repair, (middle) GOBP cellular response to DNA damage stimulus, and (right) GOBP apoptotic process are enriched in LHON patient RGC cells.

CircRNAs are extensively demonstrated to function as an miRNA sponge to achieve downstream biological effects [12,14,15]. Remarkably, circRNAs have been shown to interact miRNAs and contribute to the pathogenesis of several diseases. For example, a novel circRNA CirRIMS functions as a sponge for hsa-miR-148a-5p and hsa-miR-218-5p to promote the metastasis of gastric cancer [20]. CircRNA 104348 modulates the miR-187-3p/RTKN2 axis to promote hepatocellular carcinoma progression [21]. CircRNA_0000326 promotes the progression of bladder cancer through miR338-3p to regulate proto-oncogenes [22], while circRNA CircPPP1CB suppresses bladder cancer tumorigenesis via the miR-1307-3p/SMG1 axis [23]. Based upon our observations, circRNA_0087207 serves as an upregulating circRNA in LHON patient iPSC-derived RGCs but not the carrier iPSC-derived RGCs, and the overexpression/knockdown experiments revealed that circ0087207 contributes to the high apoptosis in LHON patient iPSC-derived RGCs. Thus, we hypothesized that circRNA_0087207 may exert its actions through suppressing certain beneficial microRNAs, leading to the regulation of disease-related genes and the establishment of the microenvironment suitable for LHON progression. We used Circinteractome (https://circinteractome.nia.nih.gov/; original version, Maryland, USA; accessed date: 30 January 2020) to screen the candidate miRNAs of hsa-circ-0087207. Considering several parameters, including the 3′ pairing stability, local adenine-uracil (AU) content, target site abundance, and seed-pairing stability, we ranked microRNAs by context+ score percentile and demonstrated miR-665 to have the most abundant target sites and the highest seed-pairing stability among the microRNAs, i.e., miR-1253, miR-1279, miR-5481, miR-421, miR-562, etc. Based on the analysis of circRNA-miRNA interaction, we filtered and selected the miR-665 from the top-twelve miRNAs (Figure 6A). The pairing at the seeding regions between miR-665 and circRNA_0087207 shows multiple hydrogen bonds (Figure 6B). Next, we used gene ontology to predict the downstream mRNAs and corresponding biological functions regulated by miR-665. In the gene ontology of mRNAs targeted by miR-665, the results showed that several biological functions related to exacerbated ROS production were enriched in the LHON patient iPSC-derived RGCs, including the notch signaling (Odds Ratio: 3.00), UV response upregulation (OR: 1.57), unfolded protein response (OR: 1.39), inflammatory response (OR: 1.34), and apoptosis (OR: 1.24) (Figure 6C). We next attempted to identify the core mediator among these biological pathways in patient iPSC-derived RGCs. The downstream mRNAs are supposed to exhibit the same upregulation trend similar to that of circRNA_0087207 in LHON patient iPSC-derived RGCs. To identify the direct downstream of circ_0087207-miR655 axis, we only selected the upregulated genes, rather than downregulated genes in LHON patient iPSC-derived RGCs. The heatmap showed that most upregulated genes are identified in the LHON patient iPSC-derived RGCs, rather than the carrier iPSC-derived RGCs, suggesting that this differential gene expression may contribute to the discrepancy of phenotypic manifestations in carrier iPSC-derived RGCs and LHON patient iPSC-derived RGCs (Figure 6D). All of the downstream candidate mRNAs were then subjected to the gene set enrichment analysis (GSEA). Based on the results of GSEA, the downstream enriched genes that are targeted by circRNA_0087207/miR665 in LHON patient iPSC-derived RGCs are primarily associated with several biological processes, including DNA repair, cellular response to DNA damage stimulus, and apoptotic process (Figure 6E). Together, our bioinformatic approaches indicated that hsa_circ_0087207 acts as the sponge of miR665 to regulate gene expression involved in DNA damage and repair and apoptotic cell death.

## 4. Discussion

Over the years, several efforts have been made to explore the therapeutic options and the disease mechanisms of LHON. For example, idebenone has been approved as the drug used for treating LHON patients due to its antioxidant properties and electron transfer that can overcome the mitochondrial complex I respiratory chain deficiency and prevent the progression of LHON [24,25,26]. This disease has also been indicated to involve mitophagy activation [27,28]. In addition, the toxicity of some toxic substances (e.g., cigarettes) may worsen the severity of LHON [29]. However, despite the progress of this research, the treatment efficacy of current therapeutic options for LHON has remained generally inefficient and blurred. The LHON unaffected carriers have been known to carry the same mitochondrial mutation but tend not to develop the disease phenotypes and the clinical manifestations of LHON. The discrepancy of phenotypes between the carriers and LHON patients suggested that certain factor(s) besides the mitochondrial mutation determine the disease feature of LHON.

As a maternally inherited mitochondrial disease, LHON is characterized by the mutations in the genes responsible for the mitochondrial electron transport chain complex I subunit. We previously generated LHON patient iPSC-derived RGCs and unaffected carrier iPSC-derived RGCs and demonstrated the defective neural outgrowth in patient iPSC-derived RGCs but impaired antioxidant enzyme expression and high ROS levels in both patient-derived cells and unaffected carrier-derived cells [30,31]. In these studies, patient iPSC- and carrier iPSC-derived RGCs as ideal in vitro platforms provided remarkable data showing the distinct phenotypes between the iPSC-derived RGCs derived from LHON patients and unaffected carriers [30,31]. In the present study, we further demonstrated that LHON patient iPSC-derived RGCs also exhibited defective firing patterns and abnormal resting membrane potential under patch-clamp recording. CircRNAs carry circular and stable structures and have therefore been employed as potential biomarkers of neurodegenerative diseases [16]. Using next-generation sequencing and circinteractome, we identified circRNA_0087207 as a crucial circRNA highly upregulated in LHON patient iPSC-derived RGCs but not in carrier iPSC-derived RGCs. Overexpression and knockdown of circRNA_0087207 revealed that this circRNA potentially serves a role in the exacerbated apoptosis but not the high ROS levels in patient iPSC-derived RGCs. To our knowledge, there has not been any literature about the circ_0087207. ALDH1A, the parent gene of circ_0087207, belongs to the aldehyde dehydrogenase family, which is one of the main enzymes involved in alcohol metabolism. This gene encodes the cytosolic isozyme, one of the two major isozymes of this enzyme. Studies in mice show that, through its role in retinol metabolism, ALDH1A gene may also be involved in the regulation of the metabolic responses to high-fat diet feeding. In this study, we used CircInteractome to show that circRNA_0087207 suppresses miR665 to stimulate a variety of downstream target genes, and these downstream genes that are enriched in LHON patient iPSC-derived RGCs are primarily associated with DNA damage and repair and apoptotic cell death.

Although it has been reported that enhancing mitochondrial ROS production is the cause of apoptosis upon mitochondrial complex I inhibition [32], our findings of high ROS levels in unaffected carrier iPSC-derived RGCs suggested that ROS might be necessary but not insufficient for the development of cellular dysfunction and apoptosis in LHON pathogenesis. One possible interpretation could be that ROS serves as an upstream regulator in the pathologic process from circ_0087207. Remarkably, our data revealed the high expression levels of circ_0087207 in LHON patient iPSC-derived RGCs (Figure 4). Manipulation of circ_0087207 indicated that this circRNA predominantly contributed to RGC apoptotic cell death in LHON (Figure 5). In addition, miR665, the downstream miRNA with the highest affinity to circ_0087207, is inhibited by circ_0087207 to positively regulate DNA damage/repair and apoptosis (Figure 6). These findings supported that this circ0087207/miR665 axis is likely crucial for the development of LHON and may explain the discrepancy of disease phenotypes in the iPSC-derived platform and the clinical observations between LHON patients and unaffected carriers. However, considering LHON as a rare disease, the recruitment of subjects remains a challenge across LHON-related studies. It should be noted that these results are obtained from the comparison between a healthy subject, an unaffected carrier, and a single patient with a single G11778A mutation, from the same family. The findings should be interpreted in this context, and further evidence will be necessary and worthwhile to shed light on the disease mechanism and potential therapeutic strategies of LHON.

Despite the development of medication (e.g., Idebenone) against LHON through suppressing the ROS generation or improving electron transfer, its high price and inconsistent efficacy raised the urgent need for novel therapeutic options [24,25,26]. Gene therapy that delivers the ND4 gene to the patients holds promises in LHON patients, but its sustained efficacy and safety remain unclear in clinical trials [33]. In this study, we proposed that circ_0087207 could potentially be used as the disease biomarker of LHON, and targeting the circ_0087207/miR665 axis may provide opportunities to rescue RGC death in LHON patients. LHON is characterized by the mitochondrial ND4 mutation and the major cause of this disease is accepted to be mitochondrial complex I deficiency. Thus, as a mitochondrial complex I inhibitor, rotenone has been used to induce apoptosis and mitochondrial ROS production, thereby widely employed for the induction of LHON-like symptoms in murine [32,34,35]. However, there are only four circular forms of ALDH1A1 that include hsa_circ_0087207 in humans but nine circular forms of the same gene in mice. Among these nine identified circular forms, so far, there is no murine circRNA that is identical to hsa_circ_0087207 or carries similar functions. Together, the lack of mitochondrial ND4 mutation and the diversity of murine ALDH1A1 circRNAs hinder the investigation of the exact causal effect between mitochondrial mutation and the downstream manifestations of LHON phenotypes in mice. Further preclinical studies are required to examine the efficacy of circ_0087207/miR665 targeting in LHON patients who carry mitochondrial ND4 mutation.

## Figures and Tables

**Table 1:** Sequences of primers used for qRT-PCR analysis.

Gene Name	Forward Sequence	Reverse Sequence
ND4	ATGACTCCCTAAAGCCCATG	CAGAGAGTTCTCCCAGTAGGTTA
ND6	CCGAGCAATCTCAATTACAATA	TTTCTGTTGAGTGTGGGTTTAG
ND1	TAAAGTCCTACGTGATCTGAGT	CGATGGTGAGAGCTAAGGTC
ALDH1A1	TGTTAGCTGATGCCGACTTG	AGTGTTGAGCGGGCTAAGAA
CIRC0087207 (CIRCALDH1A1)	AATTGCTATGGCGTGGTAAGTG	GGAGTTTGCTCTGCTGGTTTGA
CIRC0065113	AATTTGCTCGTCCATCTCGG	AGGCAGCAGGTTCTGTTTAGCT
CIRC0060475	GACATTTAGCCAATGCCACCAC	CGAGGATCTTCTTCAGGTAGCTCA
CIRC0049478	GCCACTCCTTGGTACAAATGGA	GCCGAAGAGACTCTTGAACTCATC
AJUBA	AGGCCAGGGAGGACTACTTC	TCTCCACTGCAGACTTCCAA
ATG4B	GGCAAGTCTATAGGCCAGTGGTA	GGCCCTGCATAACCTTCTGA
BBC3	GACGACCTCAACGCACAGTA	CACCTAATTGGGCTCCATCT
TP73	GACCGAAAAGCTGATGAGGA	TCAGCTCCAGGCTCTCTTTC
WWTR1	CAGCAATGTGGATGAGATGG	TCATTGAAGAGGGGGATCAG
YAP1	GCAGTTGGGAGCTGTTTCTC	GCCATGTTGTTGTCTGATCG
APAF1	TTCTGATGCTTCGCAAACAC	CTGGCAAATCTGCCTTCTTC
CAPN1	ACATGGAGGCCATCACTTTC	GGTCCACGTTGTTCCACTCT
CAPN2	AGGCATACGCCAAGATCAAC	CACCAGCTTCTGAAACGTGA
TP53	GTTCCGAGAGCTGAATGAGG	TCTGAGTCAGGCCCTTCTGT
TRNRSF10B	CACCAGGTGTGATTCAGGTG	CCCCACTGTGCTTTGTACCT
TRNRSF10D	CAGGAAATCCAAGGTCAGGA	CTCCTCTGACACCCTTCAGC
ATG4B	GGCAAGTCTATAGGCCAGTGGTA	GGCCCTGCATAACCTTCTGA
BCL2	ATGTGTGTGGAGAGCGTCAA	ACAGTTCCACAAAGGCATCC
MAX	ATGACATCGAGGTGGAGAGC	AGTCCCGCAAACTGTGAAAG
RASGF2	GTCTCCACCACCACACACTG	GCGTGGGTTATCGACATTCT
RASSF1	CGCAAGTTTGCACTCTTTGA	CCTTCAGGACAAAGCTCAGG
RASSF5	ACTGAGTGAAGACGGCACCT	AGGGGCAGGTAGAAGGATGT
STK4	CTGTGGGGCTGGTTCTGTAT	GTTGACCTGCTACCCCAAAA
BID	GTGTTTGGCTTCCTCCAAAG	TGCCTCTATTCTTCCCAAGC
CASP9	GAGGGAGTCAGGCTCTTCCT	TCACCAAATCCTCCAGAACC
FADD	AGCGGCCTAGACCTCTTCTC	CGTTAAATGCTGCACACAGG
GADD45B	AATCCACTTCACGCTCATCC	GACCAGGAGACAATGCAGGT
CDH1	TGCCCAGAAAATGAAAAAGG	GTGTATGTGGCAATGCGTTC
tRNA(Leu(UUR))	CAC CCA AGA ACA GGG TTT GT	TGG CCA TGG GTA TGT TGT TA
B2-microglobulin	TGC TGT CTC CAT GTT TGA TGT ATC T	TCT CTG CTC CCC ACC TCT AAG T

## Data Availability

The data presented in this study are available on request from the corresponding author.

## References

[1] Iorga R.E., Mihailovici R., Ozturk M.R., Costin D. (2018). Leber’s hereditary optic neuropathy—Case report. Rom. J. Ophthalmol..

[2] Bianco A., Bisceglia L., Trerotoli P., Russo L., D’Agruma L., Guerriero S., Petruzzella V. (2017). Leber’s hereditary optic neuropathy (LHON) in an Apulian cohort of subjects. Acta Myol..

[3] Meyerson C., Van Stavern G., McClelland C. (2015). Leber hereditary optic neuropathy: Current perspectives. Clin. Ophthalmol..

[4] Carelli V., Barboni P., Zacchini A., Mancini R., Monari L., Cevoli S., Liguori R., Sensi M., Lugaresi E., Montagna P. (1998). Leber’s Hereditary Optic Neuropathy (LHON) with 14484/ND6 mutation in a North African patient. J. Neurol. Sci..

[5] Puomila A., Viitanen T., Savontaus M.L., Nikoskelainen E., Huoponen K. (2002). Segregation of the ND4/11778 and the ND1/3460 mutations in four heteroplasmic LHON families. J. Neurol. Sci..

[6] Zhang Y., Tian Z., Yuan J., Liu C., Liu H.L., Ma S.Q., Li B. (2017). The Progress of Gene Therapy for Leber’s Optic Hereditary Neuropathy. Curr. Gene Ther..

[7] Abu-Amero K.K. (2011). Leber’s Hereditary Optic Neuropathy: The Mitochondrial Connection Revisited. Middle East Afr. J. Ophthalmol..

[8] Manickam A.H., Michael M.J., Ramasamy S. (2017). Mitochondrial genetics and therapeutic overview of Leber’s hereditary optic neuropathy. Indian J. Ophthalmol..

[9] Yu-Wai-Man P., Newman N.J. (2017). Inherited eye-related disorders due to mitochondrial dysfunction. Hum. Mol. Genet..

[10] Stein L.R., Imai S. (2012). The dynamic regulation of NAD metabolism in mitochondria. Trends Endocrinol. Metab..

[11] Karamanlidis G., Lee C.F., Garcia-Menendez L., Kolwicz S.C., Suthammarak W., Gong G., Sedensky M.M., Morgan P.G., Wang W., Tian R. (2013). Mitochondrial complex I deficiency increases protein acetylation and accelerates heart failure. Cell Metab..

[12] Panda A.C., Grammatikakis I., Munk R., Gorospe M., Abdelmohsen K. (2017). Emerging roles and context of circular RNAs. Wiley Interdiscip. Rev. RNA.

[13] Zhang X., Wang S., Wang H., Cao J., Huang X., Chen Z., Xu P., Sun G., Xu J., Lv J. (2019). Circular RNA circNRIP1 acts as a microRNA-149-5p sponge to promote gastric cancer progression via the AKT1/mTOR pathway. Mol. Cancer.

[14] Li X., Yang L., Chen L.L. (2018). The Biogenesis, Functions, and Challenges of Circular RNAs. Mol. Cell.

[15] Barrett S.P., Salzman J. (2016). Circular RNAs: Analysis, expression and potential functions. Development.

[16] Shao Y., Chen Y. (2016). Roles of Circular RNAs in Neurologic Disease. Front. Mol. Neurosci..

[17] Kristensen L.S., Hansen T.B., Veno M.T., Kjems J. (2018). Circular RNAs in cancer: Opportunities and challenges in the field. Oncogene.

[18] Al-Enezi M., Al-Saleh H., Nasser M. (2008). Mitochondrial disorders with significant ophthalmic manifestations. Middle East Afr. J. Ophthalmol..

[19] Zhang Z., Yang T., Xiao J. (2018). Circular RNAs: Promising Biomarkers for Human Diseases. EBioMedicine.

[20] Lin J., Zhang Y., Zeng X., Xue C., Lin X. (2020). CircRNA CircRIMS Acts as a MicroRNA Sponge to Promote Gastric Cancer Metastasis. ACS Omega.

[21] Huang G., Liang M., Liu H., Huang J., Li P., Wang C., Zhang Y., Lin Y., Jiang X. (2020). CircRNA hsa_circRNA_104348 promotes hepatocellular carcinoma progression through modulating miR-187-3p/RTKN2 axis and activating Wnt/beta-catenin pathway. Cell Death Dis..

[22] Chen Y., Wang D., Shu T., Sun K., Zhao J., Wang M., Huang Y., Wang P., Zheng H., Cai Z. (2021). Circular RNA_0000326 promotes bladder cancer progression via microRNA-338-3p/ETS Proto-Oncogene 1/phosphoinositide-3 kinase/Akt pathway. Bioengineered.

[23] Wang F., Zhang Y., Zhou X., Chen X., Xiang J., Fan M., Yu Y., Cai Y., Wu H., Huang S. (2021). Circular RNA CircPPP1CB Suppresses Tumorigenesis by Interacting With the MiR-1307-3p/SMG1 Axis in Human Bladder Cancer. Front. Cell Dev. Biol..

[24] Lyseng-Williamson K.A. (2016). Idebenone: A Review in Leber’s Hereditary Optic Neuropathy. Drugs.

[25] Pemp B., Mitsch C., Kircher K., Reitner A. (2021). Changes in Visual Function and Correlations with Inner Retinal Structure in Acute and Chronic Leber’s Hereditary Optic Neuropathy Patients after Treatment with Idebenone. J. Clin. Med..

[26] Carelli V., La Morgia C., Valentino M.L., Rizzo G., Carbonelli M., De Negri A.M., Sadun F., Carta A., Guerriero S., Simonelli F. (2011). Idebenone treatment in Leber’s hereditary optic neuropathy. Brain.

[27] Fan P., Xie X.H., Chen C.H., Peng X., Zhang P., Yang C., Wang Y.T. (2019). Molecular Regulation Mechanisms and Interactions Between Reactive Oxygen Species and Mitophagy. DNA Cell Biol..

[28] Sharma L.K., Tiwari M., Rai N.K., Bai Y. (2019). Mitophagy activation repairs Leber’s hereditary optic neuropathy-associated mitochondrial dysfunction and improves cell survival. Hum. Mol. Genet..

[29] Giordano L., Deceglie S., d’Adamo P., Valentino M.L., La Morgia C., Fracasso F., Roberti M., Cappellari M., Petrosillo G., Ciaravolo S. (2015). Cigarette toxicity triggers Leber’s hereditary optic neuropathy by affecting mtDNA copy number, oxidative phosphorylation and ROS detoxification pathways. Cell Death Dis..

[30] Wu Y.R., Wang A.G., Chen Y.T., Yarmishyn A.A., Buddhakosai W., Yang T.C., Hwang D.K., Yang Y.P., Shen C.N., Lee H.C. (2018). Bioactivity and gene expression profiles of hiPSC-generated retinal ganglion cells in MT-ND4 mutated Leber’s hereditary optic neuropathy. Exp. Cell Res..

[31] Yang T.C., Yarmishyn A.A., Yang Y.P., Lu P.C., Chou S.J., Wang M.L., Lin T.C., Hwang D.K., Chou Y.B., Chen S.J. (2020). Mitochondrial transport mediates survival of retinal ganglion cells in affected LHON patients. Hum. Mol. Genet..

[32] Li N., Ragheb K., Lawler G., Sturgis J., Rajwa B., Melendez J.A., Robinson J.P. (2003). Mitochondrial complex I inhibitor rotenone induces apoptosis through enhancing mitochondrial reactive oxygen species production. J. Biol. Chem..

[33] Wan X., Pei H., Zhao M.J., Yang S., Hu W.K., He H., Ma S.Q., Zhang G., Dong X.Y., Chen C. (2016). Efficacy and Safety of rAAV2-ND4 Treatment for Leber’s Hereditary Optic Neuropathy. Sci. Rep..

[34] Aoyama Y., Inagaki S., Aoshima K., Iwata Y., Nakamura S., Hara H., Shimazawa M. (2021). Involvement of endoplasmic reticulum stress in rotenone-induced leber hereditary optic neuropathy model and the discovery of new therapeutic agents. J. Pharmacol. Sci..

[35] Heitz F.D., Erb M., Anklin C., Robay D., Pernet V., Gueven N. (2012). Idebenone protects against retinal damage and loss of vision in a mouse model of Leber’s hereditary optic neuropathy. PLoS ONE.

